# LINC00958 promotes bladder cancer carcinogenesis by targeting miR-490-3p and AURKA

**DOI:** 10.1186/s12885-021-08882-6

**Published:** 2021-10-26

**Authors:** Hongtao Zhen, Peng Du, Qiang Yi, Xiaolong Tang, Tongqing Wang

**Affiliations:** 1 Department of Urology Ward 1, Zhengzhou Central Hospital Affiliated to Zhengzhou University, No. 195 Tongbai Road, Zhongyuan District, Zhengzhou, 450007 Henan China; 2 Department of Urology, Peking University Cancer Hospital, Beijing, 100142 China

**Keywords:** LINC00958, miR-490-3p, AURKA, Bladder cancer

## Abstract

**Background:**

Bladder cancer is a prevalent malignancy of the urinary system, in which long non-coding RNAs (lncRNAs) are highly associated. We aimed to elucidate the role of LINC00958 in bladder cancer.

**Methods:**

LINC00958 expression levels were measured using qRT-PCR. The interaction of LINC00958-miR-490-3p-AURKA was analyzed by luciferase, RIP, and RNA pull-down assays. The biological roles of LINC00958, miR-490-3p, and AURKA in bladder cancer cells were analyzed using CCK8, BrdU, and transwell assays.

**Results:**

Increased expression of LINC00958 and AURKA was observed in bladder cancer tissues and cell lines. Decreased LINC00958 expression repressed bladder cancer progression and downregulation of miR-490-3p accelerated bladder cancer cell progression. Moreover, LINC00958 sponges miR-490-3p to upregulate AURKA expression, thereby promoting carcinogenesis in bladder cancer cells.

**Conclusions:**

Our study revealed that LINC00958 facilitated cell proliferation and invasion, and suppressed cell apoptosis by sponging miR-490-3p and upregulating AURKA, thus inspiring a new treatment method for bladder cancer.

**Supplementary Information:**

The online version contains supplementary material available at 10.1186/s12885-021-08882-6.

## Background

Bladder cancer is the most prevalent malignancy of the urinary system, with a 3% incidence and 2.1% death rate worldwide [1]. Despite improvements in the current diagnosis and treatment of bladder cancer, drug resistance leads to a high recurrence rate [2, 3]. Therefore, it is essential to understand how bladder cancer develops and identify more effective targets for the treatment and prognosis of bladder cancer patients.

Long non-coding RNAs (lncRNAs) are non-coding RNAs containing over 200 nucleotides, which are key modulators of tumorigenesis [4–6]. LncRNAs have been shown to participate in a variety of tumor pathological processes by inhibiting miRNAs to upregulate the expression of their target genes [7] [8–10]. Long intergenic non-protein coding RNA 958 (LINC00958) is affiliated with the lncRNA class and is involved in cell growth, migration, and metastasis in some cancers, such as cervical, lung, and gastric cancer [11–13]. For instance, LINC00958 modulates cell sensitivity to radiotherapy by inhibiting miRNA-5095 and upregulating RRM2 expression in cervical cancer [11]. In bladder cancer, only one study has suggested that LINC00958 facilitated the pathological process of bladder cancer by repressing miR-378a-3p and elevating IGF1R expression [14]. Nevertheless, the regulation of lncRNAs in cancers is a complex process, and there may be other downstream factors of LINC00958 involved in the progression of bladder cancer that need further study.

miRNAs are non-coding RNAs of approximately 20 nucleotides, which typically function by degrading the expression of their target genes [15]. Dysregulation of miRNAs has been regarded as a critical factor in cancer cell growth and apoptosis in cancers such as colon, ovarian, and lung cancer [16–18]. Both miR-490-3p and miR-490-5p are members of the miR-490 family, which participate in tumorigenesis [19, 20]. Evidence has shown that miR-490-3p prevents cell growth and invasion, whereas it increases apoptosis in various cancers, such as prostate cancer [21], esophageal squamous cell carcinoma [22], and breast cancer [23]. Several studies have suggested the inhibitory action of miR-490-5p in bladder cancer [24–26]; however, no study has explored the molecular mechanism of miR-490-3p in the development of bladder cancer.

The aurora kinase A (AURKA) gene is located on chromosome 20q13.2 and consists of 12 exons. It encodes a cell cycle-regulated kinase that participates in microtubule formation [27, 28]. High-throughput data analysis demonstrated that AURKA was identified as a tumor promoter in all types of cancers [29–31]. Upregulation of AURKA has been reported in bladder cancer progression, which might be a potential therapeutic target for bladder cancer [32–34]. For example, overexpression of AURKA accelerated cell proliferation and reduced cell apoptosis, and high AURKA expression predicted poor prognosis in bladder cancer [33]. Furthermore, miRNA-124-3p inhibited cell proliferation and migration, but enhanced cell apoptosis by reducing AURKA in bladder cancer [35]. Nonetheless, the effect of LINC00958 and miR-490-3p on AURKA remains unknown.

In this study, we sought to determine the role of the LINC00958-miR-490-3p-AURKA axis in bladder cancer. We hypothesized that LINC00958 aggravates bladder cancer progression via the miR-490-3p-AURKA axis.

## Methods

### Samples and cells

Cancer tissues and adjacent normal tissues from 34 patients with bladder cancer, who provided informed written consent, were obtained from our hospital. The study was approved by the ethics committee of our hospital. The clinicopathological characteristics of the patients are listed in Table 1. Human bladder cancer cell lines (5637, RT4, T24, and UMUC3) and a normal human ureteral epithelial cell line (SV-HUC-1) were purchased from ATCC (Manassas, VA, USA). 5637 and UMUC3 cells were cultured in RPMI-1640 medium (Gibco, Waltham, MA, USA), RT4 and T24 cells were cultured in McCoy’s 5A medium (Gibco), and SV-HUC-1 cells were cultured in Ham’s F-12 K medium (Gibco) with 10% FBS (Gibco) at 37 °C with 5% CO_2_.

**Table 1:** Clinicopathological characteristics of bladder cancer

Clinicopathological characteristics	N (%)
Total	34
Age (year)	
>60	19 (55.88)
≤ 60	15 (44.12)
Sex	
Male	27 (79.41)
Female	7 (20.59)
Smoking	
Yes	16 (47.06)
No	18 (52.94)
Grading	
G1	8 (23.53)
G2	13 (38.24)
G3	11(32.35)
G4	1 (2.94)
n.a.	1 (2.94)
Tumor size (cm)	
< 3	15 (44.12)
≥ 3	19 (55.88)
Tumor number	
1	26 (76.47)
2–7	5 (14.71)
≥ 8	1 (2.94)
Not specified	2 (5.88)
Alguria	
Yes	11 (32.35)
No	23 (67.65)

### RT-qPCR

Trizol reagent (Cat#: 15596018, Thermo Fisher Scientific, Waltham, MA, USA) was used to extract mRNA and lncRNA. The miRcute miRNA extraction kit (DP501, Tiangen, Beijing, China) was used for miRNA extraction. The PrimeScript First Strand cDNA Synthesis kit (RR037A, Takara, China) was used to convert mRNA and lncRNA to cDNA. The All-in-One miRNA First Stand cDNA Synthesis Kit (AMRT-0060, GeneCopoeia, Rockville, MD, USA) was used to convert miRNA into cDNA. Finally, RT-qPCR was performed on the ABI 7500 Sequence Detection System with SYBR Premix Ex Taq (RR420A, Takara, China) for mRNA and lncRNA, and All-in-One miRNA qRT-PCR Detection Kit (AOMD-Q050, GeneCopoeia) for miRNA. The quantification of LINC00958 and AURKA relative to GAPDH, and miR-490-3p relative to U6 was performed using the formula 2^-ΔΔCt^. All primers are listed in Table 2.

**Table 2:** Primer sequences of qRT-PCR

Genes	Primer sequences
LINC00958	F:5′-AGAAGGAGGAGAAGCAA-3′
	R:5′-TGTGAAGTTGCAGGGAGGA-3′
miR-490-3p	F:5′-TGCGGTTCAAGTAATTCAGGA-3′
	R:5′-CCAGTGCAGGGTCCGAGGT-3′
AURKA	F:5′-GGAATATGCACCACTTGGAACA-3’
	R:5′-TAAGACAGGGCATTTGCCAAT-3’
GAPDH	F:5′-CATGAGAAGTATGACAACAGCCT-3’
	R:5′-AGTCCTTCCACGATACCAAAGT-3’
U6	F:5′-GTGCTCGCTTCGGCAGCACATATAC-3’
	R:5′-AAAAATATGGAACGCTTCACGAATTTG-3’

### Subcellular localization

The PARIS Kit (AM1921, Life biosciences, Boston, MA, USA) was used to extract the subcellular fractionation for the LINC00958 localization experiment. After obtaining the nuclear and cytoplasmic fractions, we detected the LINC00958 level in the nucleus and cytoplasm by RT-qPCR, where GAPDH was used as the internal control of the cytoplasmic fraction and U6 was used as the internal control of the nuclear fraction.

### Cell transfection

SiRNA LINC00958 (Si-lnc), miR-490-3p mimics, miR-490-3p inhibitor, SiRNA-AURKA, and the corresponding negative control (NC) were purchased from RiboBio (Guangzhou, China). RT4 and T24 cells were cultured in 24-well plates (2 × 10^5^ cells), and the plasmids with the final concentration of 50 nM, and 2 μl Lipofectamine 2000 (Invitrogen, Waltham, MA, USA) were added to the plates. After 48 h of incubation, the following experiments were performed.

### CCK8 assay

The CCK8 kit (Cat#: K1018; APExBIO, China) was used. RT4 and T24 cells (4 × 10^4^ cells) were cultured in 96-well plates. After 24, 48, 72, and 96 h of transfection, 10 μL of CCK8 solution was added to each well and incubated for 2 h. Finally, the OD values were quantified using a multimode plate reader at 450 nm (Thermo Fisher Scientific).

### BrdU assay

BrdU Cell Proliferation ELISA kit (Cat#: ab126556, Abcam, UK) was used to perform the BrdU assay. RT4 and T24 cells (2 × 10^5^ cells) were seeded into 96-well plates, which were then labeled with 20 μL BrdU for 12 h to incorporate BrdU into the proliferating cells. Following this, cells were fixed with a fixing solution and DNA was denatured. The cells were incubated with BrdU primary antibody for 1 h at 25 °C and then with the secondary antibody for 1 h at 25 °C. Finally, TMB solution was added to each well. After the color developed, the stop solution was added to each well. The OD value (450 nm) was quantified using a multimode plate reader (Thermo Fisher Scientific).

### Transwell assay

Transwell chambers (Cat#: #3244, Corning Inc., Corning, NY, USA) with 8 μm pores were precoated with Matrigel (2 μg/well, BD Biosciences, Franklin Lakes, NJ, USA) on the top side of the insert membrane in a 24-well plate. To the lower chamber, 600 μL of 5% FBS cell culture medium was added, and the upper chamber was seeded with 200 μL serum-free RT4 and T24 cell suspensions (5 × 10^4^ cells) and incubated for 24 h. The unattached cells were then cleaned using cotton swabs. The inserts were immobilized in methanol. After fixation, 1% crystal violet (C0775, Sigma-Aldrich, St. Louis, MO, USA) was used for dyeing (30 min). Finally, cells at the bottom of the membrane in each chamber were counted under a microscope (Olympus, Tokyo, Japan).

### Dual luciferase reporter assay

The psiCHECK2 LINC00958 wild-type vector and psiCHECK2 AURKA 3′UTR wild-type vector containing the miR-490-3p binding sites, psiCHECK2 LINC00958 mutated vector, and psiCHECK2 AURKA 3′UTR mutated vector without the miR-490-3p binding sites were constructed by GenePharma (Shanghai, China). RT4 and T24 cells were cultured in 24-well plates (2 × 10^5^ cells), and the cells were co-transfected with negative control (NC) or miR-490-3p mimic using Lipofectamine 3000 (Invitrogen). After 72 h of incubation, the Dual-Luciferase Reporter Assay System was used (Cat#: E1910, Promega, San Luis Obispo, CA, USA) to detect firefly and renilla luciferase activities. The firefly activity was normalized to the renilla activity.

### RIP assay

The Magna RIP RNA-binding protein immunoprecipitation kit (Cat#: 17–700, Millipore, Burlington, MA, USA) was used to detect the interaction between LINC00958 and miR-490-3p. The prepared cell lysates of RT4 and T24 cells transfected with miR-490-3p mimic or NC mimic were immunoprecipitated with magnetic beads conjugated with Ago2 (Cat#: ab186733, Abcam) or the control IgG (Cat#: ab172730, Abcam) for 4 h at 4 °C. Finally, the precipitates were collected, and the enrichment of LINC00958 was subjected to RT-qPCR.

### RNA pull-down analysis

The RT4 and T24 cells were transfected with biotin-labeled miR-490-3p (Bio-miR-490-3p) and biotin-labeled negative control (Bio-NC) (RiboBio) for 24 h. Following this, cell lysates were obtained and incubated with streptavidin beads (Cat#: #88817, Thermo Fisher Scientific) for 2 h at 25 °C. Then, the bound RNAs were eluted and purified using the RNeasy Mini Kit (Cat# 74104, Qiagen, Germany). Finally, AURKA expression was determined using RT-qPCR.

### Western blotting (WB) analysis

Whole-cell lysates of RT4 and T24 cells were treated with RIPA buffer (Cat#: #20–188, Sigma-Aldrich). Then, 30 μg of proteins were separated on 12% SDS-PAGE gels and transferred to PVDF membranes. After blocking with 5% BSA in TBST and washing twice with TBST, the membranes were incubated with anti-Bax (1:1000, Cat#: #ab32503, Abcam), anti-Bcl-2 (1:1000, Cat#: #ab32124, Abcam), anti-AURKA (1:1000, Cat#: #14475, Cell Signaling Technology (CST), Danvers, MA, USA), and anti-GAPDH (1:2000, Cat#: #5174, CST) antibodies overnight at 4 °C. Then, the membranes were incubated with the secondary antibody anti-HRP rabbit (1:10000, Cat#: 7074, CST) for 1 h. ECL reagents (P0018S, Beyotime, China) were used to obtain protein bands. The relative expression of AURKA was normalized to GAPDH and analyzed using the Image Lab software (Bio-Rad, Hercules, CA, USA).

### Statistical analysis

Student’s t-test for two groups and one-way analysis of variance (ANOVA) for multiple group comparisons were performed using GraphPad Prism 8.0 (GraphPad Prism, USA). Pearson’s correlation analysis was used to detect the association between LINC00958 and miR-490-3p or AURKA and miR-490-3p. *P* < 0.05 was considered statistically significant. Data are shown as mean ± standard deviation (SD).

## Results

### The selection of LINC00958-miR-490-3p-AURKA axis as our study object

**Fig. 1:**
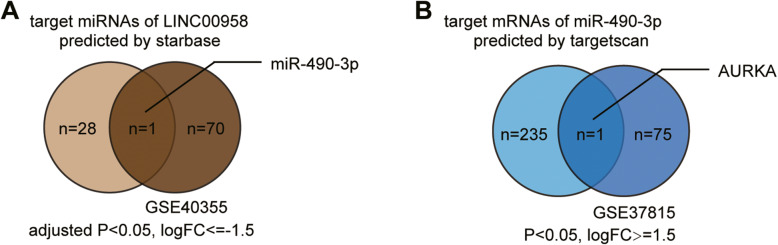
The identification of miR-490-3p and AURKA as the downstream network players of LINC00958. **A** A Venn diagram showing the intersection of the predicted target miRNAs of LINC00958 by starbase (http://starbase.sysu.edu.cn/) and the differentially expressed miRNAs of GSE40355 (selection criteria: adjusted *P* < 0.05, logFC ≤ − 1.5). **B** A Venn diagram showing the intersection of the target mRNAs of miR-490-3p predicted by targetscan (version 7.2 of human database) and the differentially expressed mRNAs of GSE37815 (selection criteria: adjusted *P* < 0.05, logFC ≥1.5)

LINC00958 has been extensively studied in some cancers such as head and neck cancers [36–38], gastric cancer [13], lung cancer [12], and cervical cancer [11, 39]*.* It is also a potential oncogenic factor and a candidate prognostic biomarker in bladder cancer [40, 41] and is associated with lymphangiogenesis and lymphatic metastasis [42]. Only Cui et al. reported LINC00958 in a ceRNA network in bladder cancer [14]. Thus, LINC00958 has been rarely studied in bladder cancer. To expand the downstream network of LINC00958, we first analyzed the GSE40355 and GSE37815 data series to identify differentially expressed miRNAs (DE-miRs) and differentially expressed mRNAs (DE-mR) in bladder cancer samples. By intersecting the predicted target miRNAs of LINC00958 using starBase and the DE-miR list (adjusted *P* < 0.05, logFC ≤ − 1.5), we identified miR-490-3p (Fig. 1A). The cancer-suppressive role of miR-490-3p has been demonstrated in various cancers [43–46], but has not been studied in bladder cancer. Thus, we speculated that LINC00958 promotes bladder cancer malignancy by suppressing miR-490-3p. We sought to identify the potential downstream network of LINC00958-miR-490-3p. By intersecting the DE-mR list of GSE37815 (*P* < 0.05, logFC ≥1.5) and the target mRNAs of miR-490-3p predicted by TargetScan (version 7.2 of human database), we identified AURKA (Fig. 1B). AURKA has also been reported to be a significant facilitator in bladder cancer [33, 34] and has been studied in miRNA-mRNA interaction networks [35]. However, AURKA has not been previously studied in a ceRNA network involving an lncRNA in bladder cancer. Thus, we hypothesized that AURKA might be regulated by LINC00958-miR-490-3p, thereby affecting the malignant phenotypes of bladder cancer.

### LINC00958 is upregulated in bladder cancer tissues and cells

**Fig. 2:**
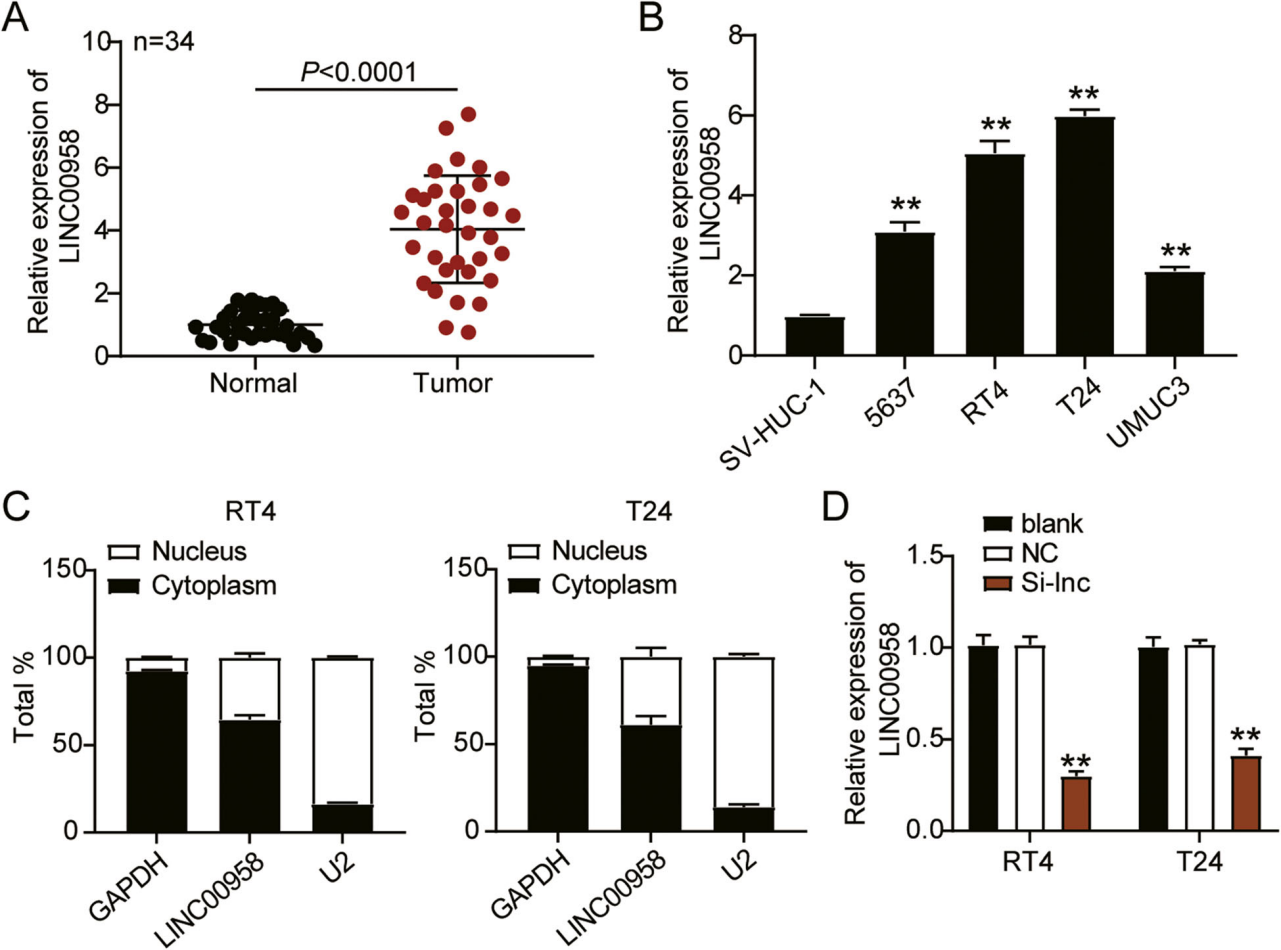
LINC00958 was upregulated in bladder cancer tissues and cells. **A** RT-qPCR detection of LINC00958 expression in bladder cancer tissues (*n* = 34) and normal tissues (*n* = 34). **B** Measurement of LINC00958 expression in bladder cancer cell lines (5637, RT4, T24, and UMUC3) and normal human ureteral epithelium cell line (SV-HUC-1). **C** The localization of GAPDH, LINC00958, and U2 in the cytoplasm and nucleus were detected in RT4 and T24 cell lines by RT-qPCR. **D** RT-qPCR analysis of LINC00958 in RT4 and T24 cells transfected with NC and Si-lnc. ^*^, *P* < 0.05; ^**^, *P* < 0.001. blank, blank control; NC, negative control; Si-lnc, siRNA-LINC00958

To analyze the biological function of LINC00958 in bladder cancer, LINC00958 expression in bladder cancer tissues and cells was first determined, which was dramatically elevated 4-fold in bladder cancer tissues compared to normal tissues (Fig. 2A). Furthermore, we also analyzed the correlation between LINC00958 expression and certain clinical parameters such as sex, age, smoking, tumor size, tumor grade, tumor number, and pathological T stage, and found that increased LINC00958 expression was correlated with tumor grade (*P* = 0.020) and pathological T stage (*P* = 0.039) in bladder cancer ​(Table 1). LINC00958 expression in bladder cancer cell lines (5637, RT4, T24, and UMUC3) was significantly elevated by more than 2-fold compared to that in the normal human ureteral epithelium cell line (SV-HUC-1). Notably, the RT4 and T24 cell lines showed the highest LINC00958 expression; thus, we chose them for subsequent experiments (Fig. 2B). Next, we observed that 60% of LINC00958 was localized in the cytoplasm, while 40% of LINC00958 was found in the nucleus, suggesting that LINC00958 mainly exists in the cytoplasm (Fig. 2C). Then, we transfected siRNA-LINC00958 (Si-lnc) and negative control (NC) into RT4 and T24 cells and confirmed the silencing efficiency. As displayed in Fig. 2D, LINC00958 expression was reduced by over 50% in the Si-lnc groups compared to that in the blank groups of RT4 and T24 cells. These results indicated that LINC00958 was upregulated in bladder cancer tissues and cells.

### LINC00958 enhances the progression of bladder cancer cells

**Fig. 3:**
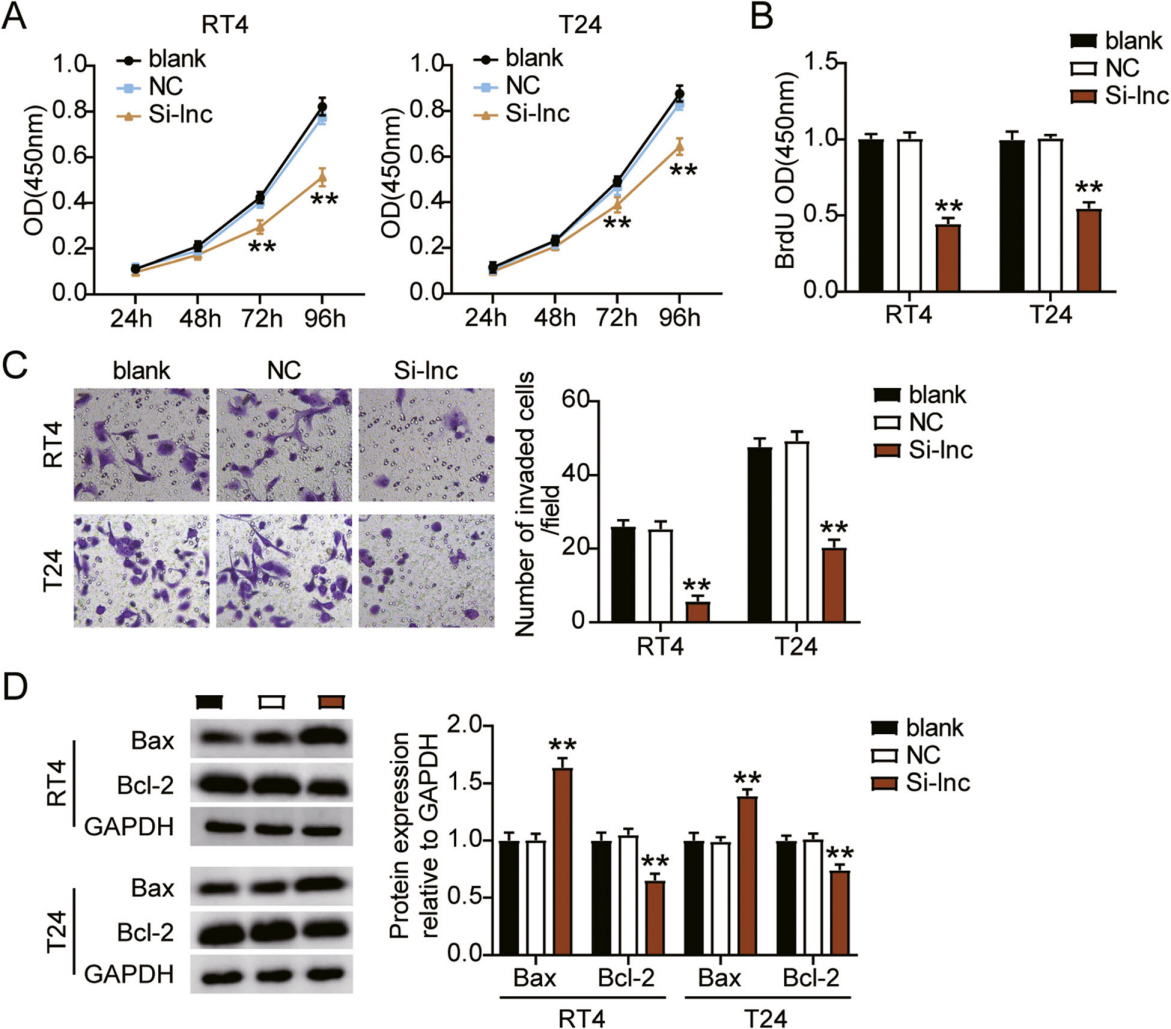
LINC00958 enhanced the progression of bladder cancer cells. (**A**) Cell viability was detected in RT4 and T24 cells transfected with NC and Si-lnc by CCK8 assay. (**B**) Cell proliferation was detected in RT4 and T24 cells transfected with NC and Si-lnc by BrdU assay. (**C**) Cell invasion ability was determined in RT4 and T24 cells transfected with NC and Si-lnc by transwell assay. (**D**) Cell apoptosis related-proteins were determined in RT4 and T24 cells transfected with NC and Si-lnc by western blotting. ^*^, *P* < 0.05; ^**^, *P* < 0.001. blank, blank control; NC, negative control; Si-lnc, siRNA-LINC00958

To investigate whether LINC00958 plays a key role in bladder cancer, we performed a series of experiments in cells transfected with Si-lnc. The CCK8 assay showed that the cell viability levels in the Si-lnc group were significantly lower than those in the blank group of both RT4 and T24 cells (Fig. 3A). Meanwhile, the BrdU assay showed that cell proliferation levels in the Si-lnc group were dramatically decreased by 50% compared to that in the blank groups of RT4 and T24 cells (Fig. 3B). Subsequently, we checked the invasion ability of the cells; cell invasion levels in Si-lnc group were significantly downregulated by 80% in the RT4 cells and by 50% in the T24 cells compared to that in the blank group (Fig. 3C). Moreover, the expression of the pro-apoptotic protein Bax was dramatically increased in the Si-lnc group compared to the blank group of RT4 and T24 cells, while the anti-apoptotic protein Bcl-2 showed the opposite tendency (Fig. 3D). Thus, these results indicate that LINC00958 enhances cell proliferation and invasion, but suppresses apoptosis of bladder cancer cells.

### LINC00958 sponges miR-490-3p in bladder cancer cells

**Fig. 4:**
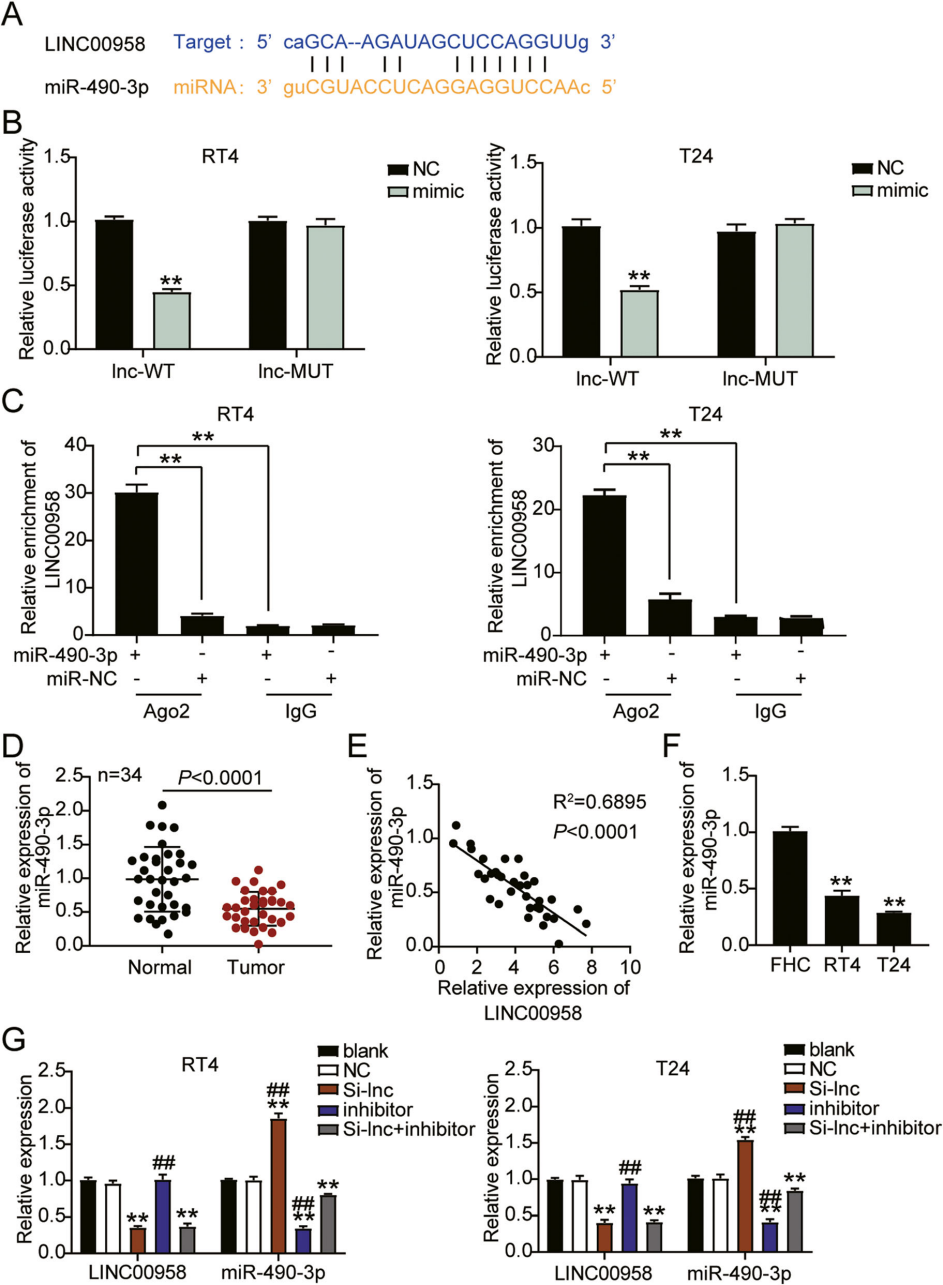
LINC00958 sponged miR-490-3p in bladder cancer cells. **A** TargetScan showed the predicted binding sequences of miR-490-3p for LINC00958. **B** Dual luciferase assay was performed in cells co-transfected with plasmids LINC00958-Wt or LINC00958-Mut and NC mimics or miR-490-3p mimic in RT4 and T24 cells. **C** RT-qPCR detection of LINC00958 expression on enrichment of miR-490-3p by RIP assay. **D** RT-qPCR detection of miR-490-3p expression in bladder cancer tissues (*n* = 34) and normal tissues (*n* = 34). **E** Correlation analysis between miR-490-3p expression and LINC00958 expression. **F** RT-qPCR detection of miR-490-3p expression in SV-HUC-1, RT4, and T24 cell lines. **G** RT-qPCR detection of LINC00958 and miR-490-3p expression in RT4 and T24 cells transfected with NC, Si-lnc, inhibitor, and Si-lnc + inhibitor. ^*^, *P* < 0.05; ^**^, *P* < 0.001 compared to blank. ^#^, *P* < 0.05; ^##^, *P* < 0.001 compared to Si-lnc + inhibitor. Blank, blank control; NC, negative control; Wt, wild-type; Mut, Mutant; Si-lnc, siRNA-LINC00958; inhibitor, miR-490-3p inhibitor

Next, we determined the interaction between LINC00958 and miR-490-3p. The binding site sequences of LINC00958 and miR-490-3p were predicted using starBase (Fig. 4A). Next, we detected the interaction by luciferase activity assay, where the miR-490-3p mimics or negative control (NC), and psiCHECK2 LINC00958-wide type (lnc-WT) or psiCHECK2 LINC00958 mutant (lnc-MUT) were co-transfected into RT4 and T24 cells. The results showed that only the co-transfection of miR-490-3p mimic and lnc-WT group showed a 50% decrease in luciferase activity, suggesting that miR-490-3p indeed interacts with LINC00958 (Fig. 4B). We also found elevated levels of LINC00958 in cells overexpressing miR-490-3p by interacting with AGO2, suggesting that LINC00958 had coordinated with miR-490-3p in both RT4 and T24 cells (Fig. 4C). Additionally, as shown in Fig. 4D, we observed that the miR-490-3p expression was 50% lower in bladder cancer tissues than in adjacent normal tissues, and there was a negative correlation between LINC00958 and miR-490-3p levels in bladder cancer tissues (Fig. 4E). Furthermore, the miR-490-3p levels in RT4 and T24 cells were downregulated by over 50% compared to that in SV-HUC-1 cells (Fig. 4F). We further transfected siRNA-LINC00958 (Si-lnc), negative control (NC), and miR-490-3p inhibitor into RT4 and T24 cells. We found that the miR-490-3p level in the inhibitor groups was 70% lower than that in the blank groups; silencing LINC00958 led to a 1.5-fold increase in miR-490-3p levels and the miR-490-3p levels in the Si-lnc + inhibitor groups were similar to those in the blank groups (Fig. 4G). Thus, miR-490-3p is a downstream gene of LINC00958 in bladder cancer cells.

### LINC00958 sponging miR-490-3p facilitates progression of bladder cancer

**Fig. 5:**
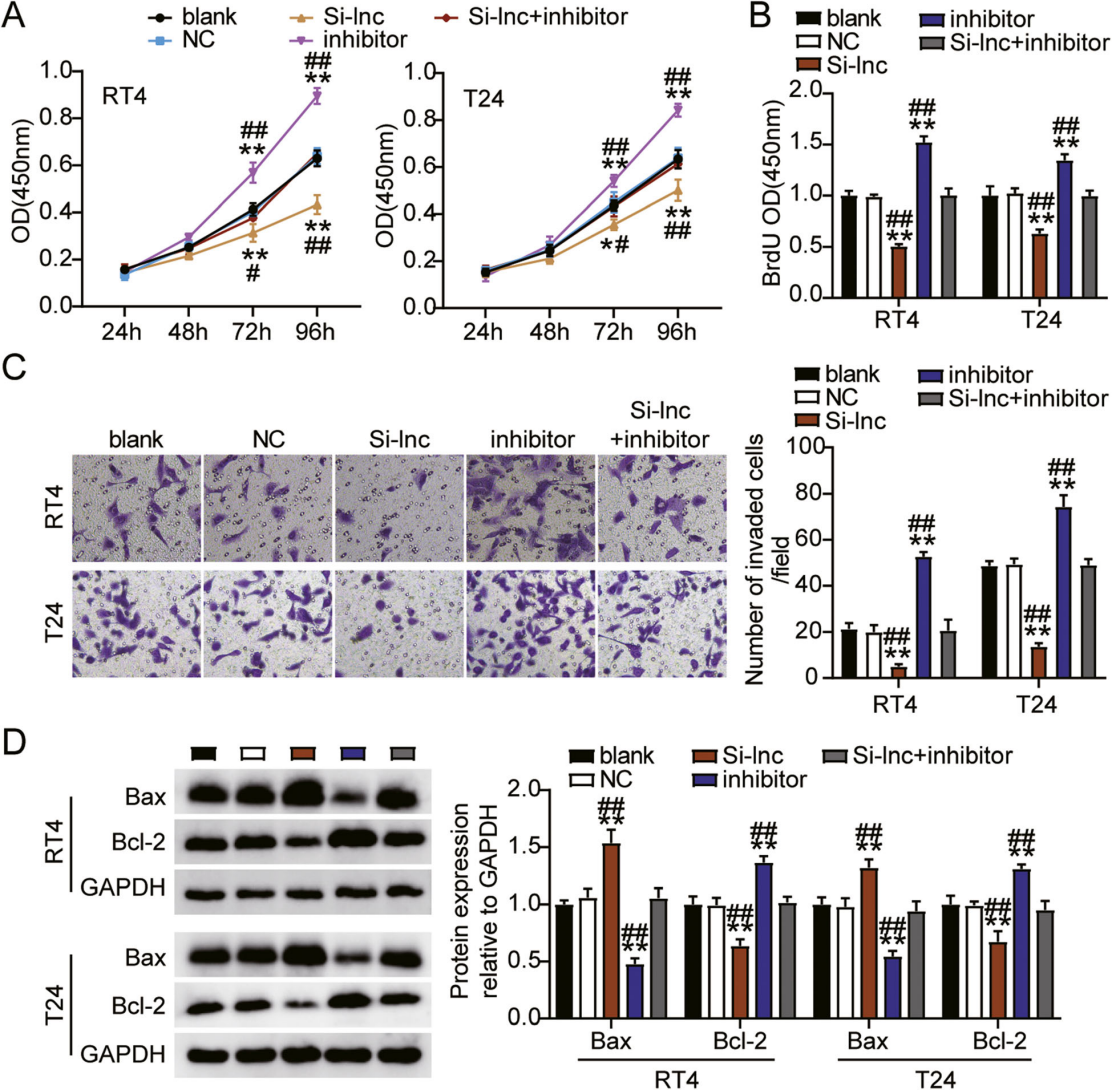
LINC00958 sponging miR-490-3p facilitated bladder cancer cell progression. **A** Cell viability was detected in RT4 and T24 cells transfected with NC, Si-lnc, inhibitor, and Si-lnc + inhibitor. **B** Cell proliferation was detected in RT4 and T24 cells transfected with NC, Si-lnc, inhibitor, and Si-lnc + inhibitor. **C** Cell invasion was detected in RT4 and T24 cells transfected with NC, Si-lnc, inhibitor, and Si-lnc + inhibitor. **D** Cell apoptosis related-proteins were detected in RT4 and T24 cells transfected with NC, Si-lnc, inhibitor, and Si-lnc + inhibitor by western blotting. ^*^, *P* < 0.05; ^**^, *P* < 0.001 compared to blank. ^#^, *P* < 0.05; ^##^, *P* < 0.001 compared to Si-lnc + inhibitor. Blank, blank control; NC, negative control; Si-lnc, siRNA-LINC00958; inhibitor, miR-490-3p inhibitor; Si-lnc + inhibitor, siRNA-LINC00958 + miR-490-3p inhibitor

We further assessed whether LINC00958 regulates miR-490-3p to affect the progression of bladder cancer. First, we found that the cell viability in the miR-490-3p inhibitor group was significantly increased, while the cell viability in the Si-lnc + inhibitor group was similar to the blank group of both RT4 and T24 cells (Fig. 5A). In addition, cell proliferation in the miR-490-3p inhibitor group was elevated by 1.5-fold, while the cell proliferation in the Si-lnc + inhibitor group was similar to that in the blank group (Fig. 5B). Furthermore, the results of the transwell invasion assay showed that the cell invasion level in the miR-490-3p inhibitor group was significantly enhanced in RT4 and T24 cells, while the Si-lnc + inhibitor group presented an invasion level similar to that of the blank group of both RT4 and T24 cells (Fig. 5C). Moreover, cell apoptosis presented by the expression of pro-apoptotic protein Bax was significantly reduced in the miR-490-3p inhibitor group, while the Bax expression level in the Si-lnc + inhibitor group was similar to that of the blank groups of RT4 and T24 cells (Fig. 5D). Collectively, LINC00958 sponging miR-490-3p facilitates bladder cancer cell progression.

### MiR-490-3p targets AURKA in bladder cancer cells

**Fig. 6:**
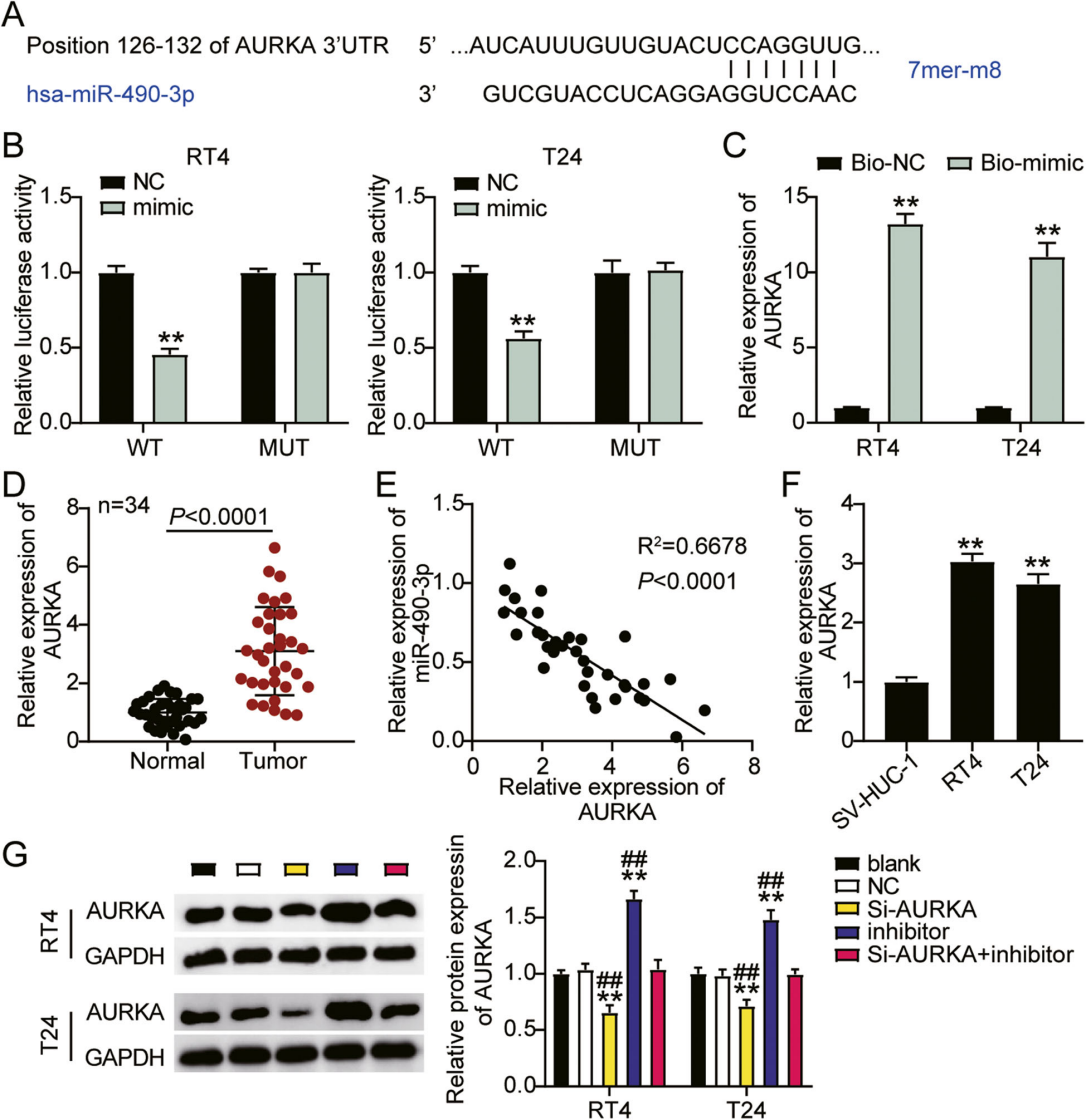
MiR-490-3p targeted AURKA in bladder cancer cells. **A** TargetScan showed the predicted binding sequences of AURKA for miR-490-3p. **B** Dual-luciferase assay was performed in cells co-transfected with plasmids AURKA 3′UTR-Wt or AURKA 3′UTR-Mut and NC mimics or miR-490-3p mimic in RT4 and T24 cells. **C** Enrichment of miR-490-3p and AURKA analyzed by RNA pull-down assay. **D** RT-qPCR detection of AURKA expression in bladder cancer tissues (*n* = 34) and normal tissues (*n* = 34). **E** The correlation between the relative expression level of miR-490-3p and AURKA. **F** RT-qPCR detection of AURKA expression in SV-HUC-1, RT4, and T24 cell lines. **G** Measurement of AURKA protein expression in RT4 and T24 cells transfected with NC, Si-AURKA, inhibitor, and Si-AURKA+inhibitor. ^*^, *P* < 0.05; ^**^, *P* < 0.001 compared to blank. ^#^, *P* < 0.05; ^##^, *P* < 0.001 compared to Si-AURKA +inhibitor. NC, negative control; Wt, wild-type; Mut, Mutant; Si-AURKA, siRNA-AURKA; inhibitor, miR-490-3p inhibitor; Si-AURKA+inhibitor, siRNA-AURKA+ miR-490-3p inhibitor

Subsequently, we sought to elucidate the interaction between miR-490-3p and AURKA. The binding site sequences of AURKA and miR-490-3p were predicted by TargetScan 7.2 (Fig. 6A). We further confirmed the interaction by luciferase activity assay, which showed that the co-transfection of miR-490-3p mimics and psiCHECK-2 AURKA 3′UTR WT plasmid showed a 50% decrease in luciferase activity compared to the NC groups, suggesting that miR-490-3p had a direct interaction with AURKA (Fig. 6B). Next, an RNA pull-down assay confirmed that miR-490-3p could bind to AURKA (Fig. 6C). In addition, miR-490-3p levels were enhanced by 3-fold in the tumor tissues compared to the normal tissues (Fig. 6D), and were negatively correlated with AURKA in bladder cancer tissues (Fig. 6E). Moreover, miR-490-3p was upregulated by approximately 3-fold in RT4 and T24 cells compared to SV-HUC-1 cells (Fig. 6F). Next, we treated RT4 and T24 cells with siRNA-AURKA (Si-AURKA), miR-490-3p inhibitor (inhibitor), and negative control (NC) to study the role of AURKA in bladder cancer. The siRNA-AURKA groups showed a 30% decrease in AURKA protein expression, while the AURKA protein expression in the inhibitor group was increased by nearly 1.3-fold when compared to that in the blank group. The levels observed in the Si-AURKA+inhibitor groups were similar to those in the blank groups (Fig. 6G). Taken together, it can be concluded that miR-490-3p directly targets AURKA in bladder cancer cells.

### MiR-490-3p targeting AURKA hampers bladder cancer cells progression

**Fig. 7:**
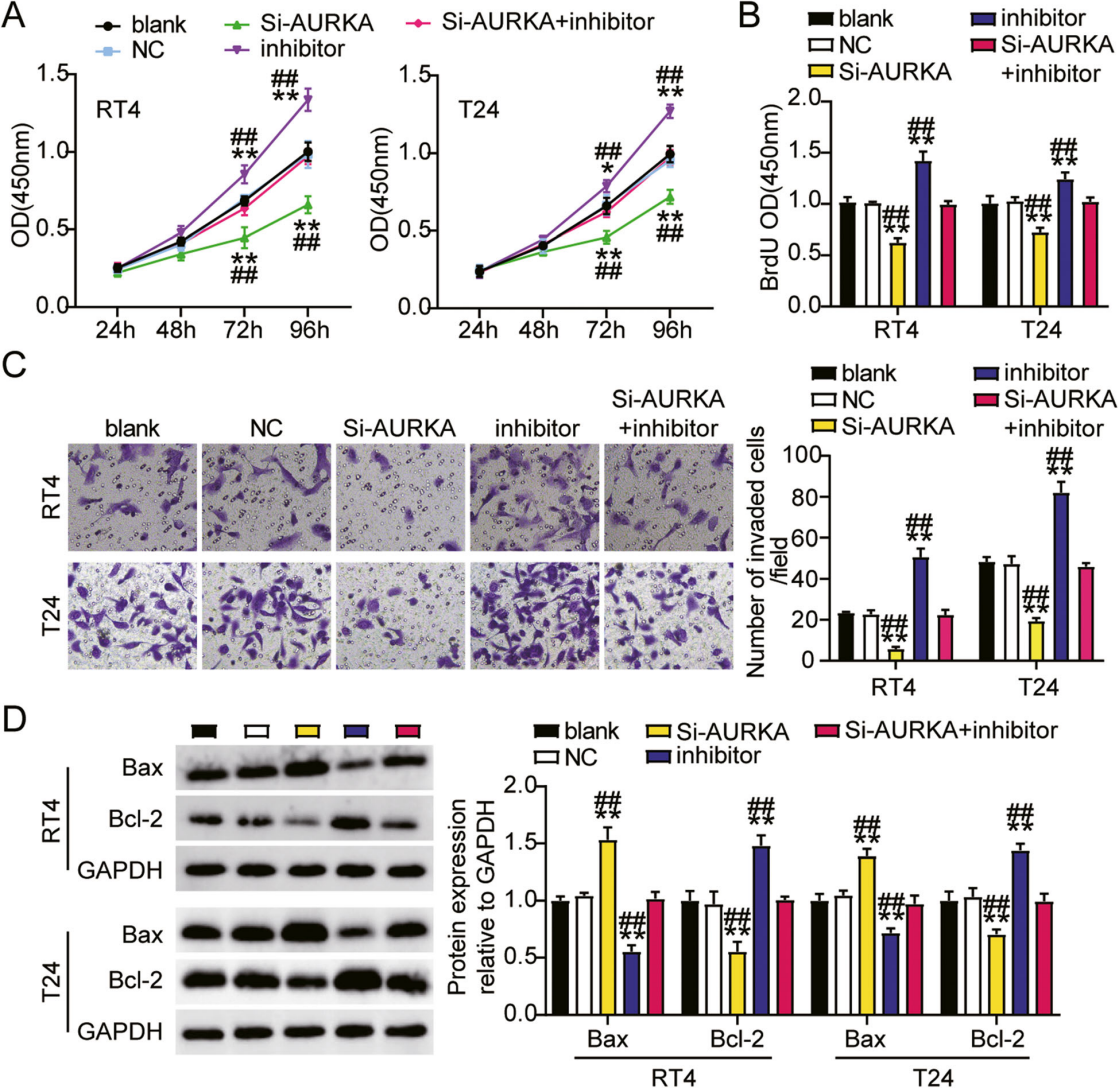
MiR-490-3p targeting AURKA hampered bladder cancer cells progression. **A** Cell viability was detected in RT4 and T24 cells transfected with NC, Si-AURKA, inhibitor, and Si-AURKA+inhibitor. **B** Cell proliferation was detected in RT4 and T24 cells transfected with NC, Si-AURKA, inhibitor, and Si-AURKA+inhibitor. C Cell invasion was detected in RT4 and T24 cells transfected with NC, Si-AURKA, inhibitor, and Si-AURKA+inhibitor. D Cell apoptosis related-proteins were detected in RT4 and T24 cells transfected with NC, Si-AURKA, inhibitor, and Si-AURKA+inhibitor by western blotting. ^*^, *P* < 0.05; ^**^, *P* < 0.001 compared to blank. ^#^, *P* < 0.05; ^##^, *P* < 0.001 compared ti Si-AURKA +inhibitor. Blank, blank control; NC, negative control; Si-AURKA, siRNA-AURKA; inhibitor, miR-490-3p inhibitor; Si-AURKA+inhibitor, siRNA-AURKA+miR-490-3p inhibitor

We further confirmed the biological action of miR-490-3p on AURKA in bladder cancer. First, we found that cell viability in the siRNA-AURKA group was dramatically impaired, while the cell viability in the Si-AURKA+inhibitor group was similar to that in the blank groups of both RT4 and T24 cells (Fig. 7A). Additionally, cell proliferation in the siRNA-AURKA group decreased by 30%, while no significant difference was observed between the Si-AURKA+inhibitor group and blank groups in both RT4 and T24 cells (Fig. 7B). Moreover, the transwell invasion assay showed that cell invasion levels in the siRNA-AURKA group were decreased by 80% in the RT4 and 50% in the T24 cells, while the Si-AURKA+inhibitor groups displayed similar cell invasion levels to blank groups in both RT4 and T24 cells (Fig. 7C). Finally, cell apoptosis levels in the siRNA-AURKA groups, indicated by the expression of the pro-apoptotic protein Bax, were evidently upregulated, while the expression levels in the Si-AURKA+inhibitor groups and the blank groups in both RT4 and T24 cells were similar (Fig. 7D). Collectively, miR-490-3p suppressed bladder cancer cell proliferation and invasion, while boosting cell apoptosis by targeting AURKA.

## Discussion

Our study confirmed that the expression of LINC00958 and AURKA was upregulated in bladder cancer, while the expression of miR-490-3p was downregulated. Furthermore, LINC00958 enhanced cell growth and invasion, but suppressed apoptosis of bladder cancer cells by inhibiting miR-490-3p and upregulating AURKA expression. Thus, the LINC00958-miR-490-3p-AURKA axis may be an effective therapeutic strategy for bladder cancer.

An increasing number of studies have demonstrated the essential role of lncRNAs in bladder cancer development, such as lncRNA SNHG1, lncRNA DLX6-AS1, and lncRNA SNHG3. All these lncRNAs facilitate bladder cancer progression by enhancing cell growth and reducing cell apoptosis [47–49]. Furthermore, several studies have revealed that LINC00958 acts as an oncogenic driver in bladder cancer [14, 42, 50]. He et al. [42] demonstrated that LINC00958 promotes bladder cancer-associated lymphangiogenesis and lymphatic metastasis in vivo and in vitro. Another study revealed that silencing of LINC00958 attenuated bladder cancer progression by repressing miR-378a-3p and elevating IGF1R expression [14]. Consistent with these studies, we also found that LINC00958 was upregulated in bladder cancer tissues and cells, and LINC00958 promoted cell viability and invasion, but inhibited cell apoptosis in bladder cancer cells. Furthermore, our study showed that LINC00958 inhibited tumorigenesis in bladder cancer cells by suppressing miR-490-3p to upregulate AURKA, which was different from previous studies on LINC00958 in bladder cancer. Therefore, our study revealed a new axis involving LINC00958-miR-490-3p-AURKA in the progression of bladder cancer.

Multiple studies have suggested that miR-490-3p acts as a suppressor in most cancers, including hepatocellular carcinoma, gastric cancer, and breast cancer [51–53]. In bladder cancer, evidence has shown that miR-490-5p inhibits proliferation via G1-phase arrest of bladder cancer by targeting c-Fos [24]. Another study also revealed that upregulation of miR-490-5p led to decreased EGFR expression, hampering cell invasion in bladder cancer cells [25]. miR-490-3p and miR-490-5p are members of the family of miR-490, but the role of miR-490-3p in bladder cancer is unknown. Our study investigated the role of miR-490-3p in bladder cancer and found an obvious downregulation of miR-490-3p expression in bladder cancer. Inhibition of miR-490-3p elevated cell growth and suppressed apoptosis in bladder cancer. Additionally, miR-490-3p, sponged by LINC00958, could regulate its target gene AURKA, thereby inhibiting the progression of bladder cancer. Our study is the first to elucidate the role of miR-490-3p in bladder cancer.

Dysregulation of AURKA has been found in numerous cancers, including lung, cervical, and renal cancers, showing that it can facilitate cell growth and invasion, but represses cell apoptosis [54–56]. As referred to bladder cancer, Guo et al. [33] reported that AURKA expression was significantly elevated in bladder cancer tissues, and the high expression of AURKA presented a poor prognosis. Another study indicated that AURKA targeted by miR-124-3p could enhance proliferation and migration and impair apoptosis of bladder cancer cells [35, 53]. Consistent with previous reports, a similar molecular mechanism of AURKA in bladder cancer was revealed in our study; high level of AURKA was also observed in bladder cancer, and the downregulation of AURKA could hamper bladder cancer progression. However, to investigate the mechanism of miR-490-3p/AURKA on the proliferation, migration, and apoptosis of cancer cells, Si-AURKA and miR-490-3p inhibitor were transfected into RT4 and T24 cells. The results showed that cell proliferation and migration were increased and apoptosis was decreased after transfection with the miR-490-3p inhibitor. In addition, Si-AURKA transfection inhibited cell proliferation and migration and promoted apoptosis. Therefore, we speculated that the effect of the inhibitor and Si-AURKA on the cancer cells was offset by each other. Hence, AURKA could be targeted by miR-490-3p to affect bladder cancer progression. Finally, we further elucidated that LINC00958 facilitated bladder cancer progression via the miR-490-3p/AURKA axis. Thus, this study is the first to report that AURKA could be regulated by both LINC00958 and miR-490-3p in bladder cancer progression.

As previously reported, evidence showed that lncRNA RP11-480I12.5 promotes the progression of breast cancer cells through the miR-490-3p-AURKA-Wnt/β-catenin axis [53]. Therefore, whether the Wnt/β-catenin pathway or other pathways participate in the LINC00958/miR-490-3p/AURKA axis in bladder cancer needs further exploration. Meanwhile, the role of the LINC00958/miR-490-3p/AURKA axis in bladder cancer also needs to be proven both in vivo and clinically. In addition, this study screened the differentially expressed miRNAs and mRNAs in bladder cancer using GEO DataSets, and the targets of LINC00958, miR-490-3p, and AURKA will be further explored in the future using multiple databases such as TCGA and GEPIA.

## Conclusions

Taken together, our findings indicate that LINC00958 aggravates cell growth and invasion, but hampers apoptosis of bladder cancer cells via the miR-490-3p/AURKA axis. This may provide a new direction for the clinical treatment of bladder cancer patients.

## Supplementary Information

**Additional file 1.**

## Data Availability

The datasets used and/or analyzed during the current study are available from the corresponding author on reasonable request.

## Abbreviations

AURKA: Aurora kinase A
lnc-WT: LINC00958-wide type
lnc-MUT: LINC00958 mutant
lncRNAs: long non-coding RNAs
miRNAs: microRNAs
NC: negative control
Si-lnc: siRNA-LINC00958
SD: standard deviation
