# Supplementary material for: LINC00958 promotes bladder cancer carcinogenesis by targeting miR-490-3p and AURKA
Source: BMC Cancer. 2021 Oct 26;21:1145. doi: 10.1186/s12885-021-08882-6 (PMC8549181; doi:10.1186/s12885-021-08882-6)

RT4 BAX for Fig.3D


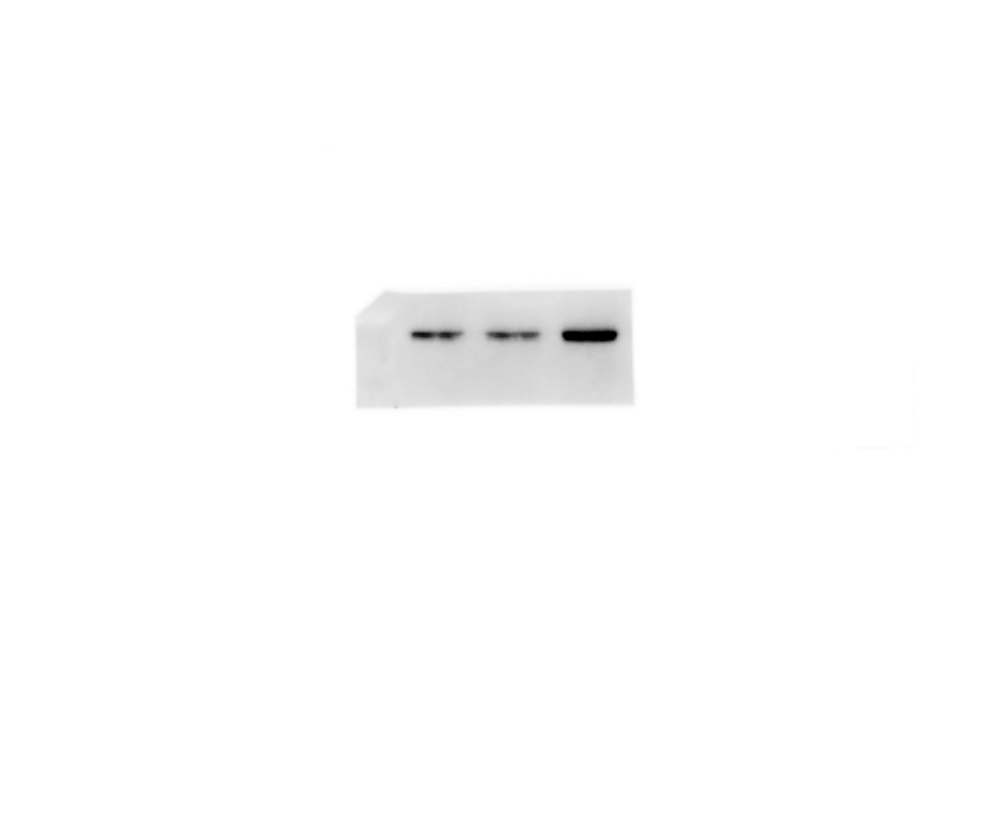


RT4 bcl-2 for Fig.3D


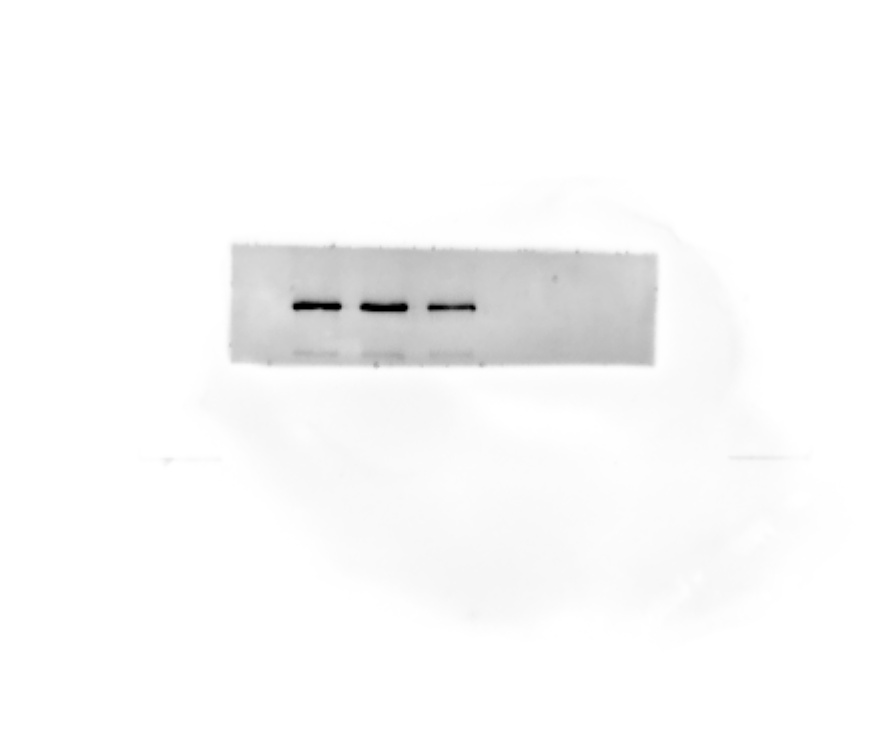


RT4 GAPDH for Fig.3D


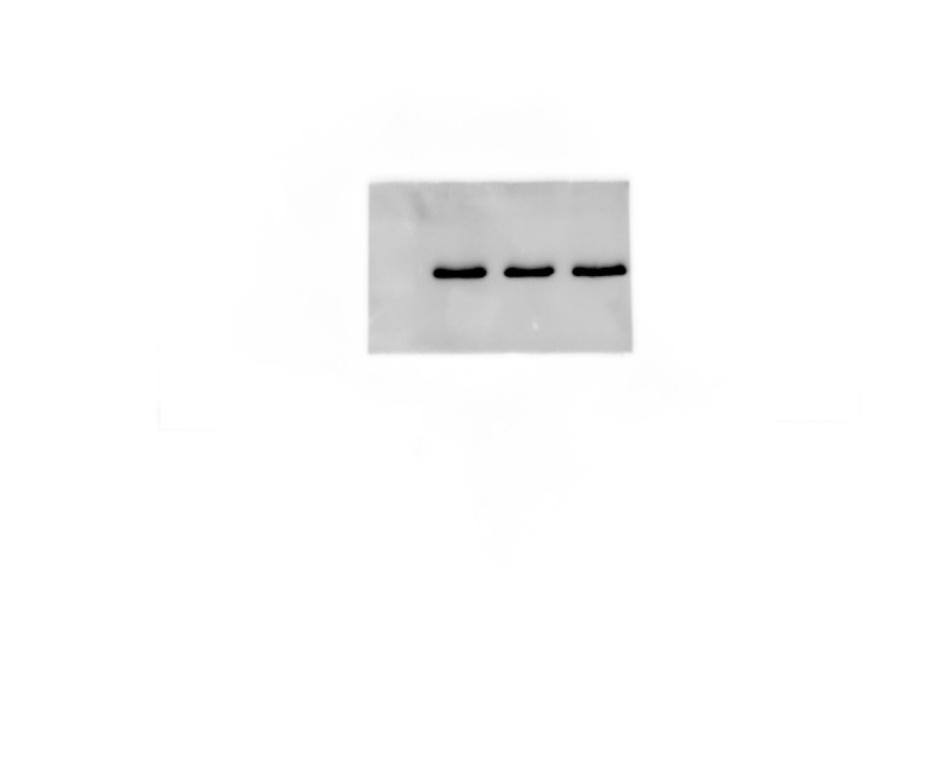


T24 bax for Fig.3D


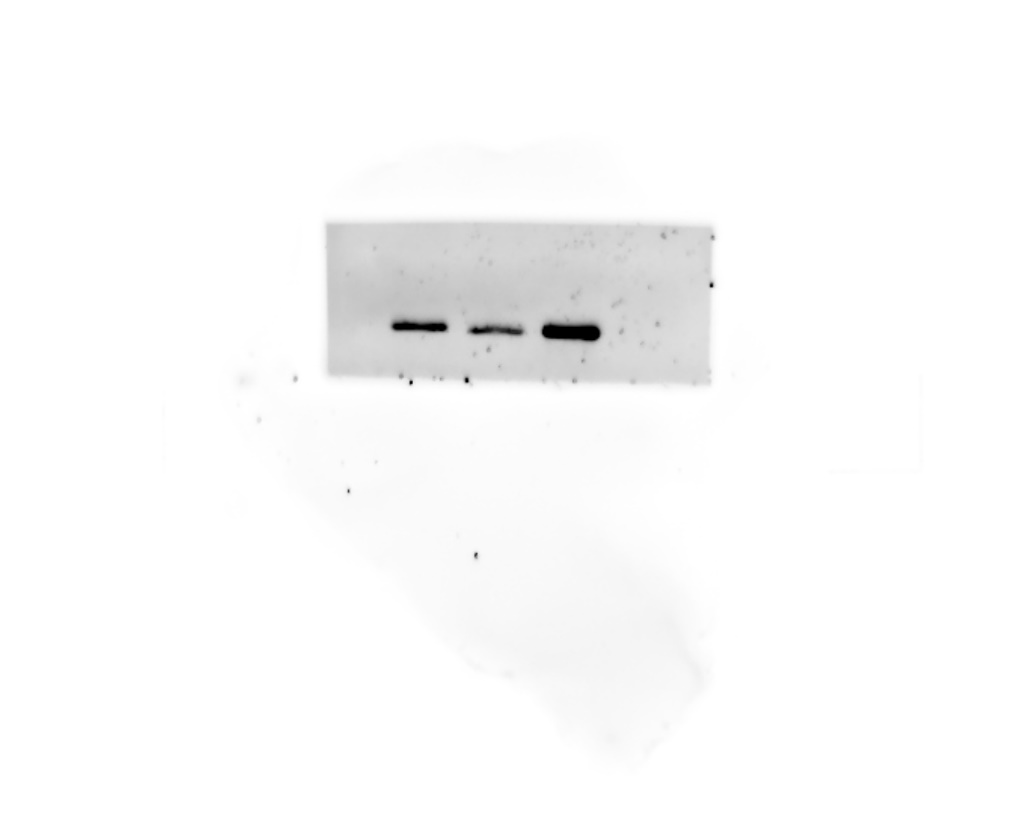


T24 bcl-2 for Fig.3D


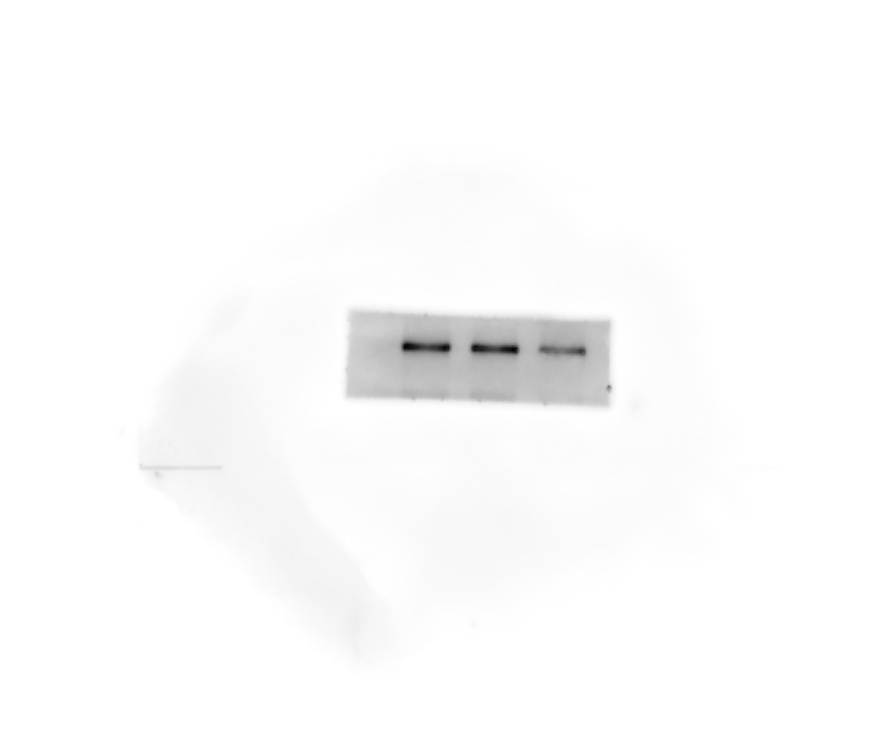


T24 GAPDH for Fig.3D


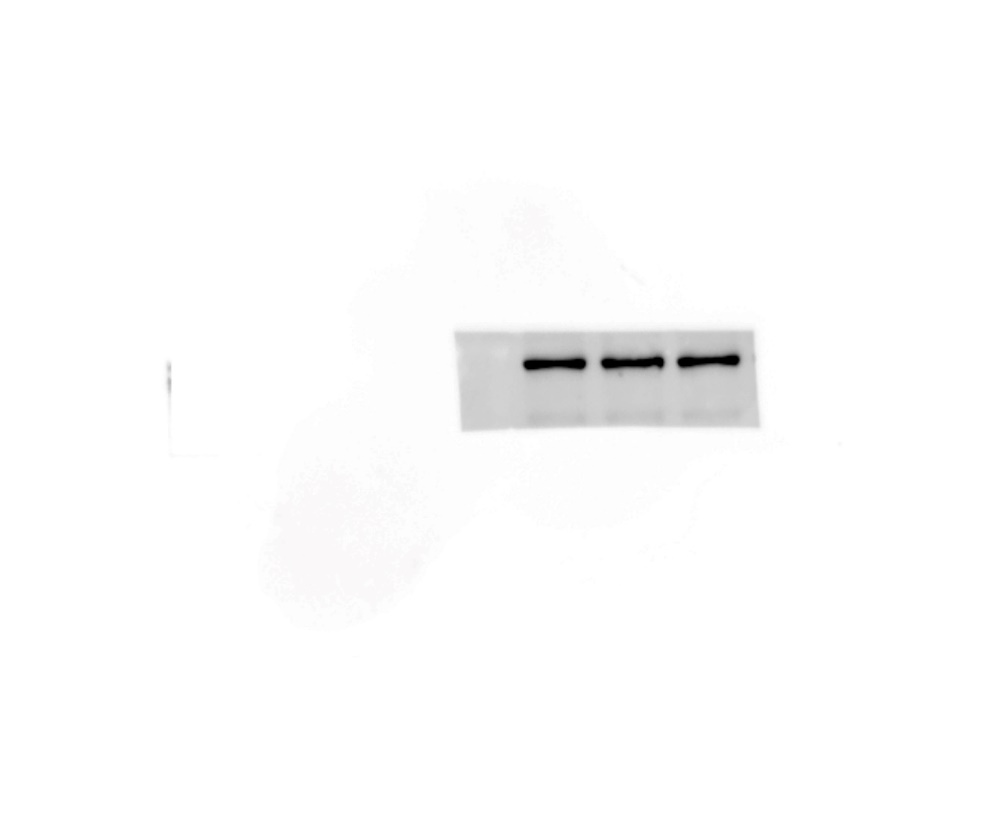


RT4 bax for Fig.5D


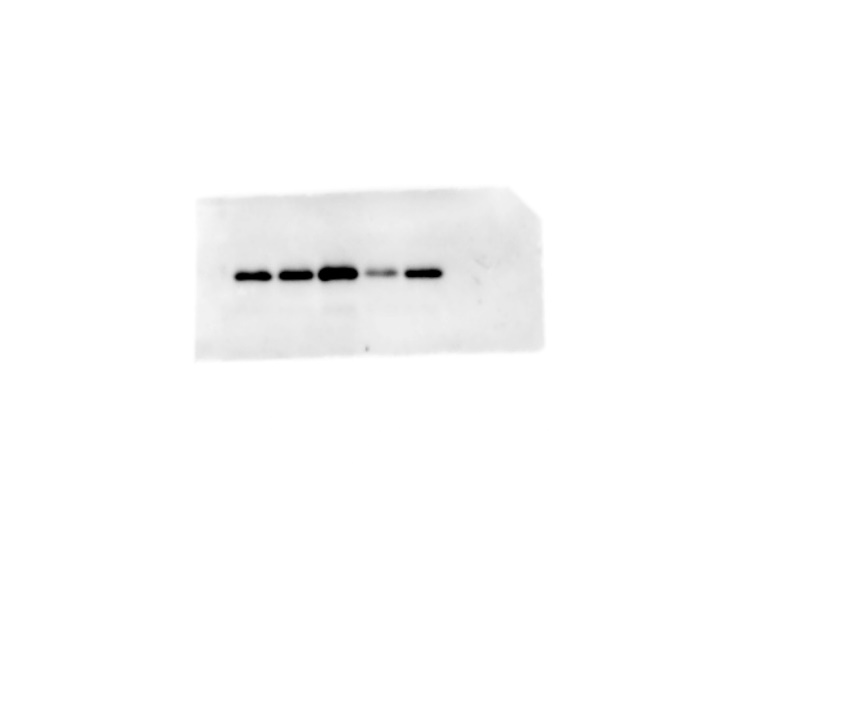


RT4 bcl-2 for Fig.5D


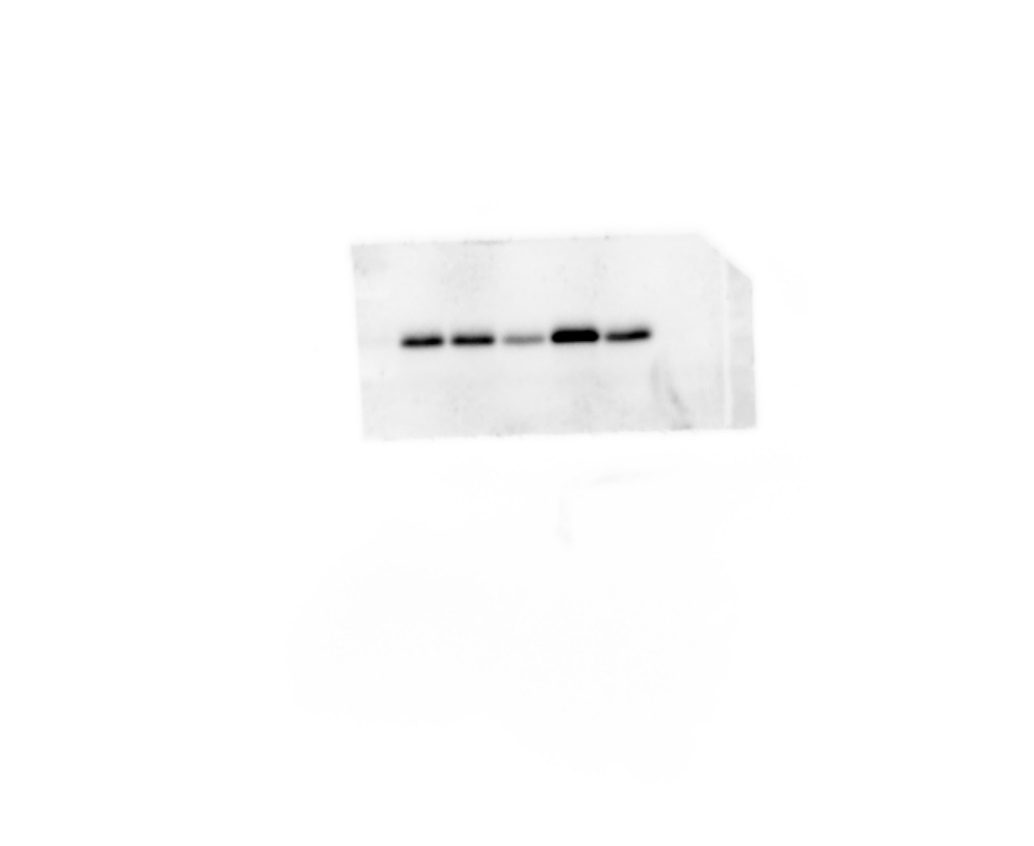


RT4 GAPDH for Fig.5D


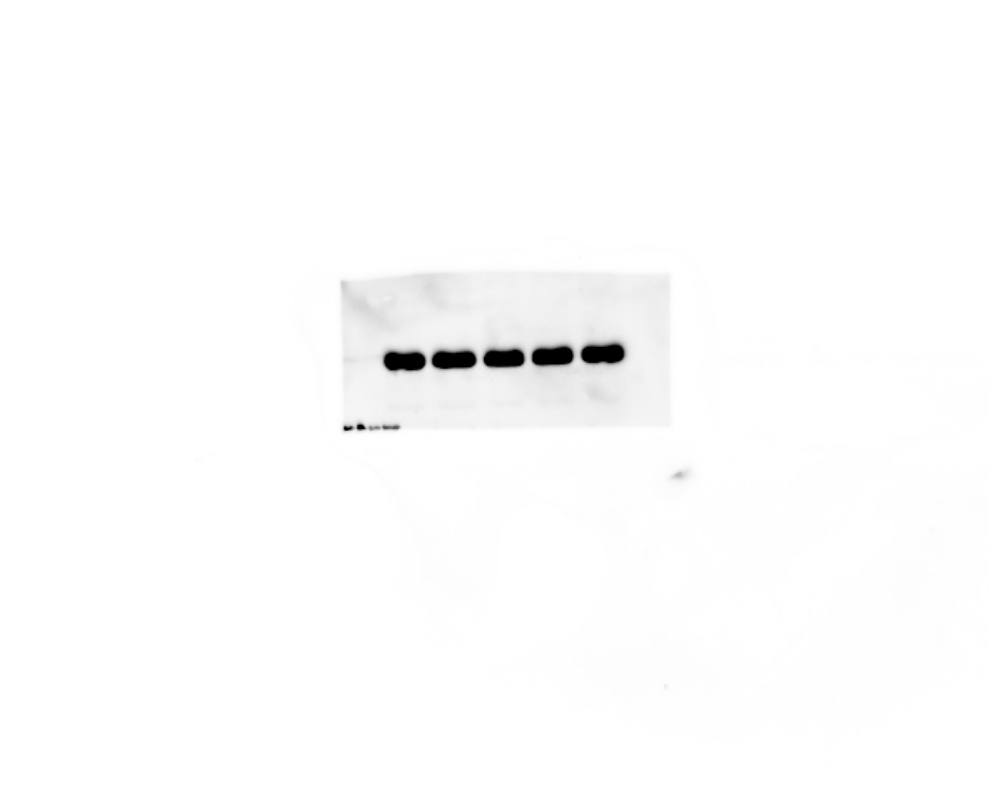


T24 bax for Fig.5D


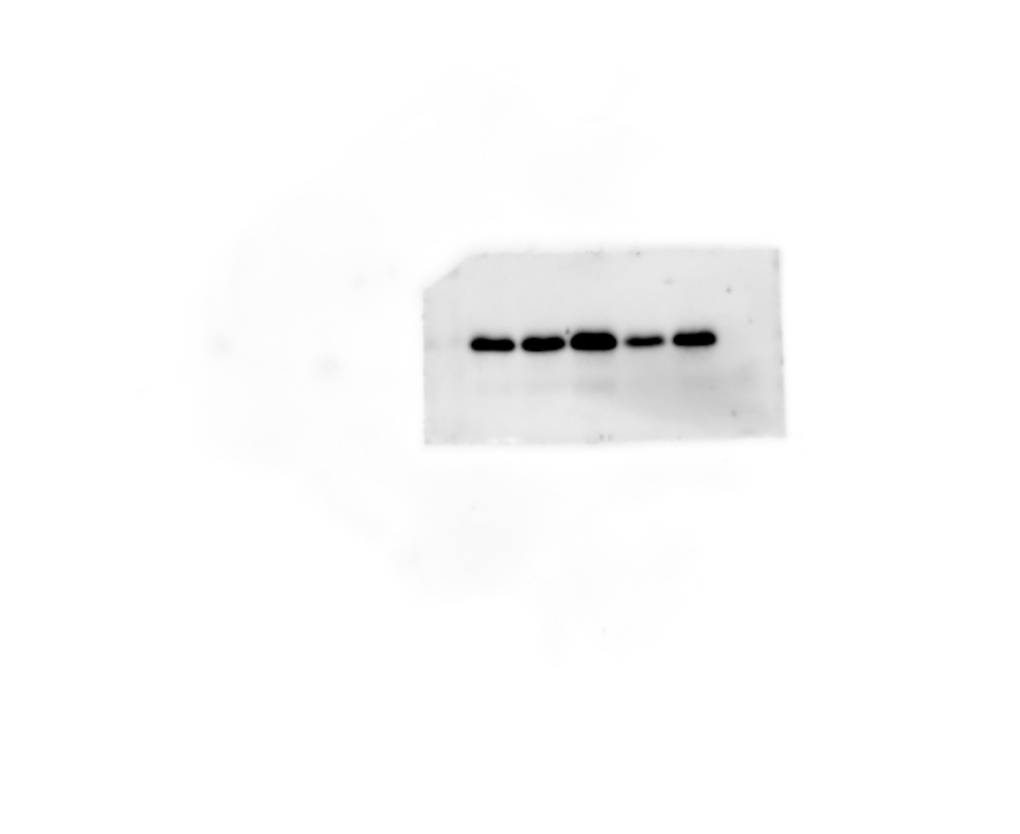


T24 bcl-2 for Fig.5D


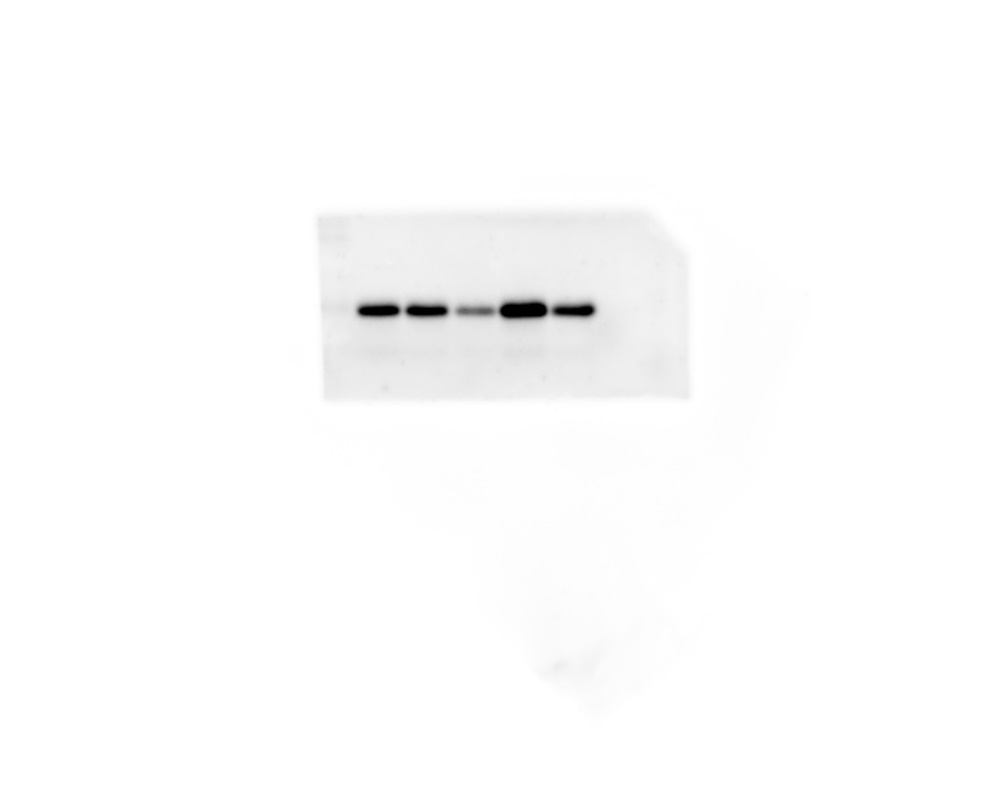


T24 GAPDH for Fig.5D


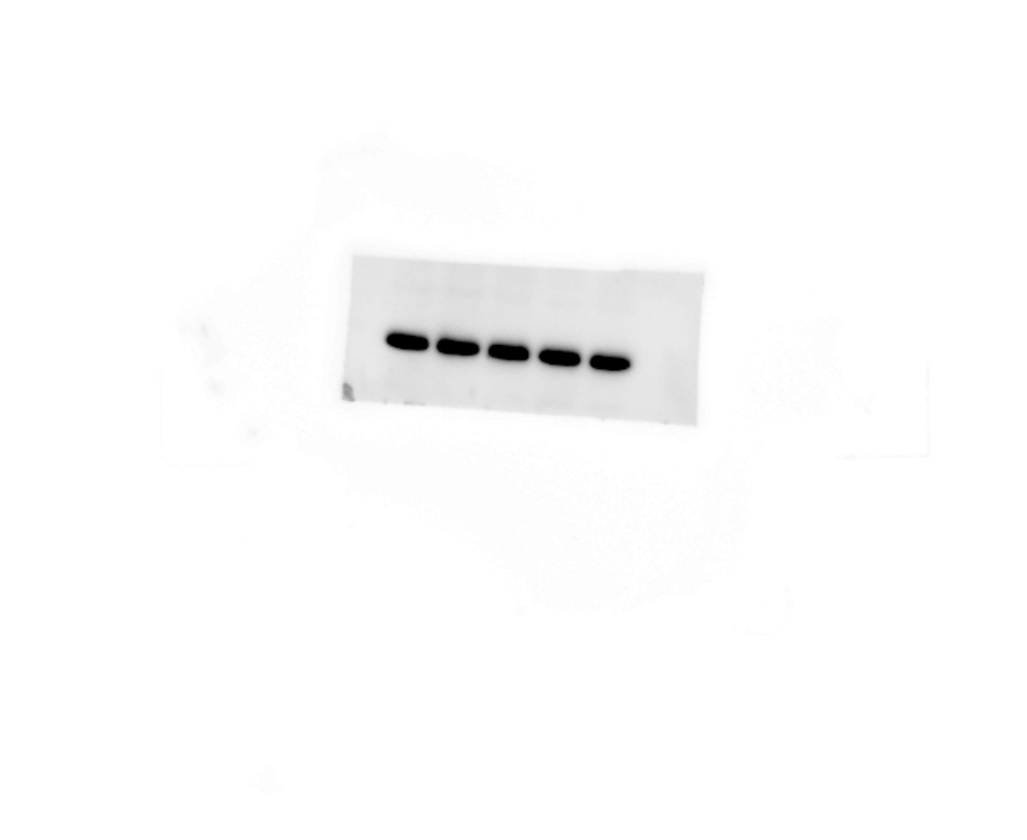


RT4 AURKA for Fig.6G


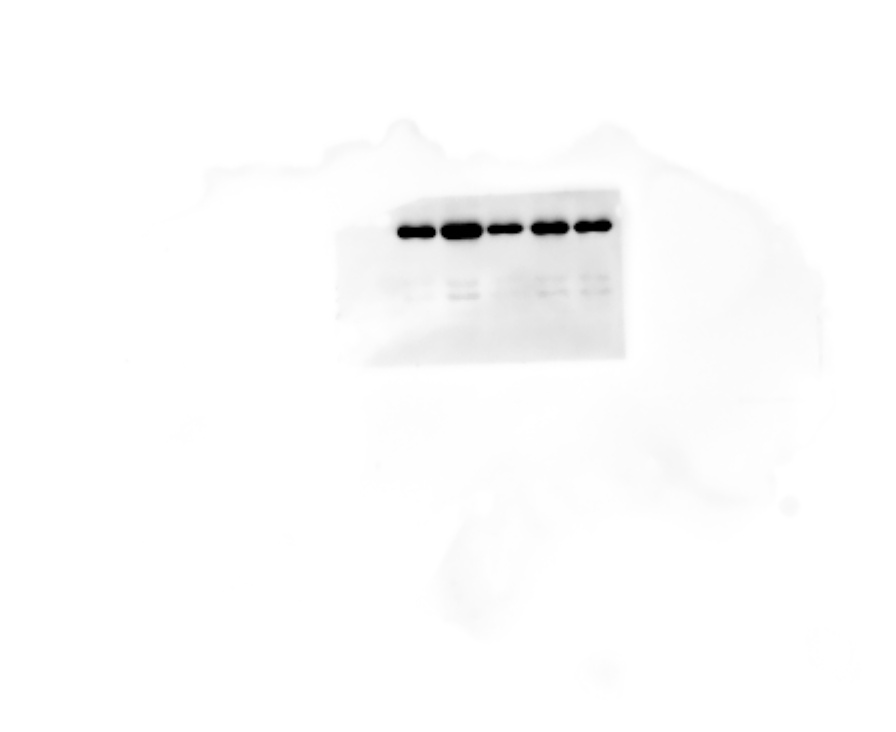


RT4 GAPDH for Fig.6G


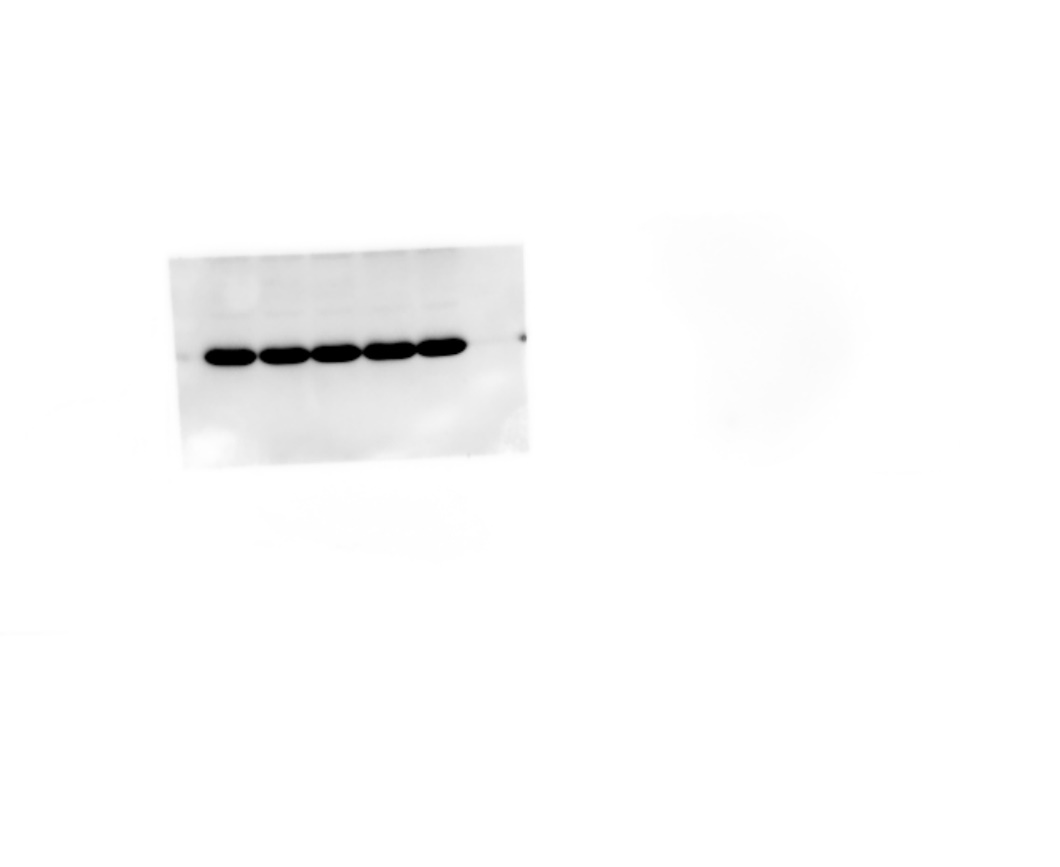


T24 AURKA for Fig.6G


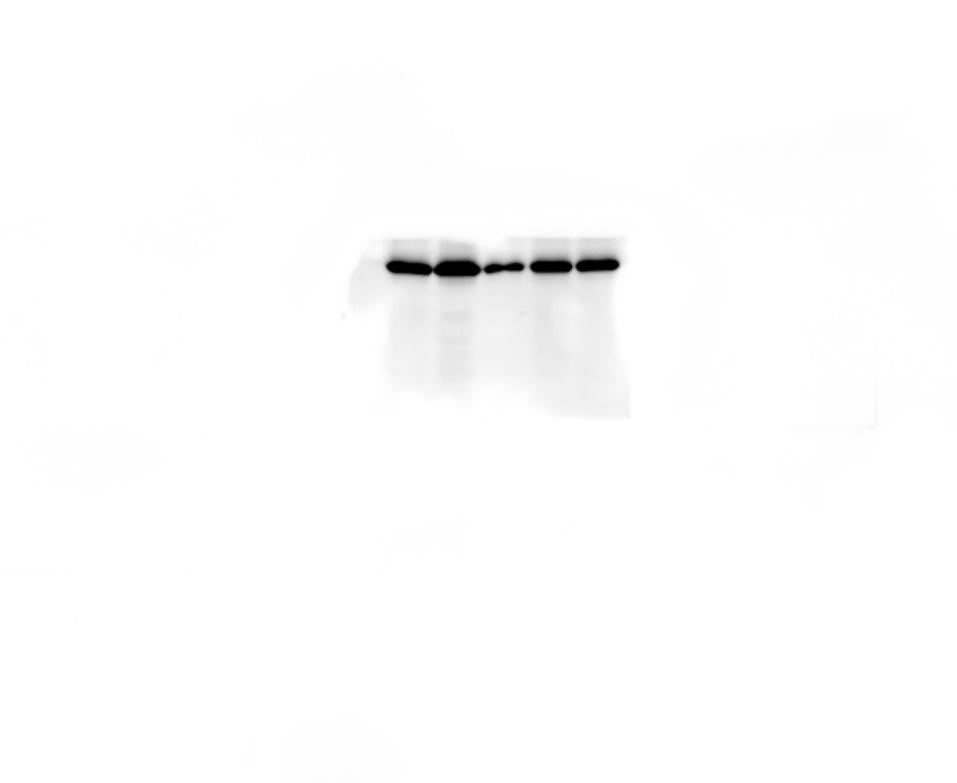


T24 GAPDH for Fig.6G


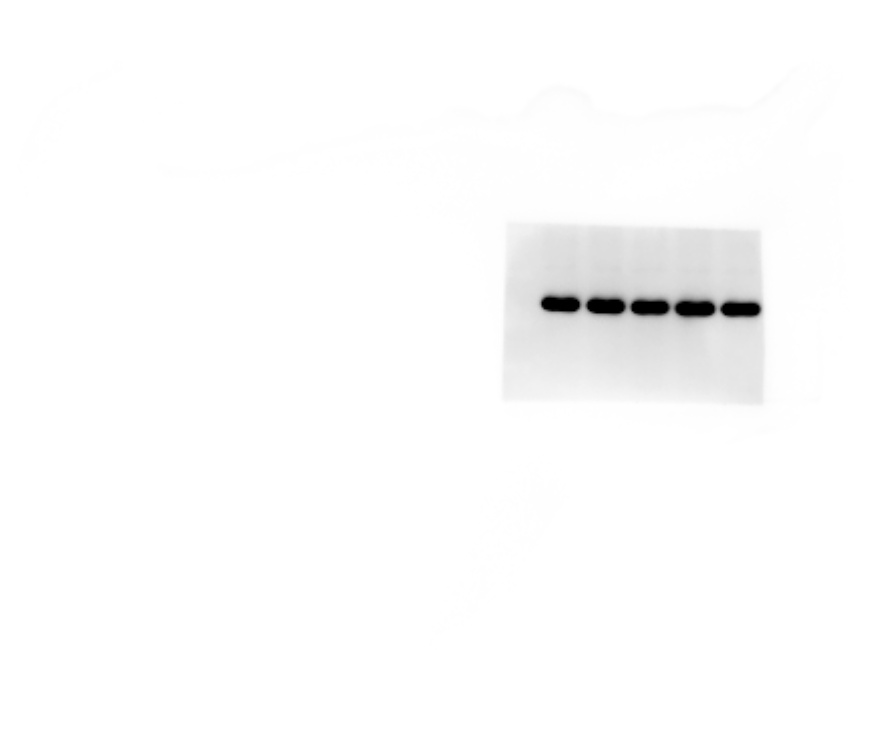

Supplement: Supplementary file 1 — Additional file 1. [file 12885_2021_8882_MOESM1_ESM.docx]
